# Analysis of the Importance of the Motion Used in the Resistance of Different Mechanical Instrumentation Systems in Endodontics: A Comparative Study

**DOI:** 10.3390/ma15134443

**Published:** 2022-06-24

**Authors:** Jesús Mena-Álvarez, Manuel Almanzor-López, Norberto Quispe-López, Ana De Pedro-Muñoz, Cristina Rico-Romano

**Affiliations:** Department of Endodontics, Faculty of Dentistry, Alfonso X El Sabio University, 28691 Madrid, Spain; manuelalmanzor1991@gmail.com (M.A.-L.); nquislop@uax.es (N.Q.-L.); amunodep@uax.es (A.D.P.-M.); cromaric@uax.es (C.R.-R.)

**Keywords:** cyclic fatigue, endodontics, Procodile, reciprocating movement, reflex smart dynamic, smart motion

## Abstract

Aim: The aim of this study was to compare the cyclic fatigue strength of different reciprocating rotary systems depending on the movement used. Methods: Four study groups were analyzed (*n* = 30): (1) Reciproc^®^, (2) Reciproc Blue^®^, (3) Wave One Gold^®^ and (4) Procodile^®^. Each group was divided into three subgroups according to the motion used: (A) Reflex Dynamic^®^ (*n* = 10), (B) ReFlex Smart^®^ (*n* = 10) and (C) conventional reciprocating motion (*n* = 10). They were used in a dynamic cyclic fatigue prototype until their fracture, and the time was measured in seconds. The results obtained were analyzed with the ANOVA method, and for two-to-two comparisons, the Tukey method and Weibull statistics were used. Results: Procodile ReFlex Smart had the longest time to failure, and statistically significant differences were found between Procodile ReFlex Smart and the other files and motions (*p* < 0.05). Conclusion: Smart motions increase cyclic fatigue strength. ReFlex Smart^®^ motion increases the cyclic fatigue strength of reciprocating rotary systems, and Procodile^®^ ReFlex Smart was the most resistant system file.

## 1. Introduction

Root canal treatment is one of the most frequently performed treatments, and its prognosis depends on a series of interrelated factors, such as chamber opening, instrumentation, disinfection and root canal filling. The main objective of root canal treatment is to promote the chemical-mechanical disinfection of the pulp cavity and its root canal system through the use of endodontic instruments and irrigation, as well as its three-dimensional obturation with an inert sealing material and a coronal seal, which prevent the entry of microorganisms [1,2,3,4,5,6,7,8,9].

The use of rotary instrumentation with nickel–titanium (NiTi) files has been a revolution in endodontic technique, as it has managed to simplify and improve the efficacy of endodontic therapy. NiTi alloy has the ability to change its atomic bond type, which causes changes in its crystallographic arrangement and mechanical properties. This alloy owes its properties to a transition between a martensitic-type structure and an austenitic-type structure [10]. Although nickel–titanium (NiTi) rotary instruments have many desirable characteristics, one of the biggest concerns and drawbacks is unexpected fracture [11]. Fractures in these instruments can occur through two mechanisms: flexural fracture and torsional fracture [12].

Torsional fracture accounts for 30–56% of fractures during clinical use, and flexural fracture accounts for 44–70%. The latter generally occurs without visible signs of permanent deformation, which makes it difficult to prevent this fracture [11]. When fractured rotary files have been analyzed under a microscope, pits, indentations and craters have been seen on the surface of the instrument [12]. The properties of NiTi instruments can be affected by manufacturing processes, chemical composition and heat treatment [13]. Although fractures of endodontic instruments depend largely on the technique and experience of clinics, numerous attempts have been made by manufacturers to improve the properties of nickel–titanium. It has been proven that, by means of heat treatment, the behavior can be modified, increasing its flexibility and achieving better properties than conventional NiTi [14,15,16]. At rest, the instruments are in the austenitic phase, and with rotary or reciprocating movement, they are in the martensitic phase, which is susceptible to deformation or fracture [17]. Initially, conventional NiTi instruments were in an austenitic phase, although later, one modification was introduced, and files were manufactured in the martensitic phase, which makes them more flexible. The introduction of this new phase of the NiTi alloy was a change and meant a new stage in the manufacture of rotary systems [18].

The movement that is performed has also been modified to incorporate smart movements. Root ZX II^®^ (J Morita, Kyoto, Japan) has two types of smart movements: the Optimum Glide Path^®^ (OGP), which is used to create a glide path in a similar way to manual movement, and the Optimum Torque Reverse^®^ (OTR), which is continuous rotation movement, but it has a mechanism that is activated when the torque exceeds a certain value, and this makes the file go back, so there is reciprocal rotation [19]. There is also adaptive motion designed for the Twisted Files^®^ (TF) system, which is continuous rotary motion and reciprocating motion. The motor has a patented algorithm, which, depending on the tension caused in the instrumentation, changes the movement of the file. Adaptive motion improves torsional fracture and cyclic fatigue resistance [20]. The last movement is Reflex^®^, which incorporates the EndoPilot^®^ motor. There are two independent movements: Reflex Dynamic^®^, which offers a higher speed and greater efficiency, and ReFlex Smart^®^, which is safer and reacts more delicately to torsional stress [21]. The motor determines the torque exerted and the torsional stress on the file, allowing the most heavily loaded area of the file to be determined. It is a movement that has a 360° rotation that is interrupted by small stops, with which it controls the torsional load and file tension. The motor adapts the movement individually to each situation; with the torque and the torsional stress, it determines which of the areas, the coronal middle or apical area, is subjected to the load and adapts the movement. Thanks to this individualization, the risk of fracture is minimized, and the use of the file is optimized. It is ideal for complex anatomies where high torsional stress occurs [21].

The aim of this work was to compare the cyclic fatigue strength of different current reciprocating rotary systems with the new smart motions ReFlex Dynamic^®^ and ReFlex Smart^®^ with their conventional reciprocating movements, in addition to knowing which file is more resistant to cyclic fatigue and with what movement.

## 2. Materials and Methods

### 2.1. Study Design

The endodontic files were randomly distributed (Epidat 4.1, Galicia, Spain) into 4 groups: 30 Procodile^®^ files (Komet Medical, Lemgo, Germany), 30 Reciproc^®^ files (VDW, Munich, Germany), 30 Reciproc Blue^®^ files (VDW, Munich, Germany) and 30 Wave One Gold^®^ files (DentsplySirona Endodontics. Ballaigues, Switzerland). Before use, they were checked with a stereomicroscope (SZR-10, Optika, Bergamo, Italy) to observe possible defects and deformities, and none were ruled out. Within each group, the endodontic files were randomized and distributed into the following subgroups: A: Reflex Dynamic Movement (*n* = 10); B: Reflex Smart Movement (*n* = 10); and C: Conventional Reciprocating Movement (*n* = 10). The sample size of the study was calculated on the basis of the EPIDAT 4.2 program (Dirección Xéral de Saude Pública, Galicia, Spain) and the article by Zubizarreta et al. [22] with statistical significance (*p*-value < 0.05).

### 2.2. The Experimental Cyclic Fatigue Model

**Figure 1:**
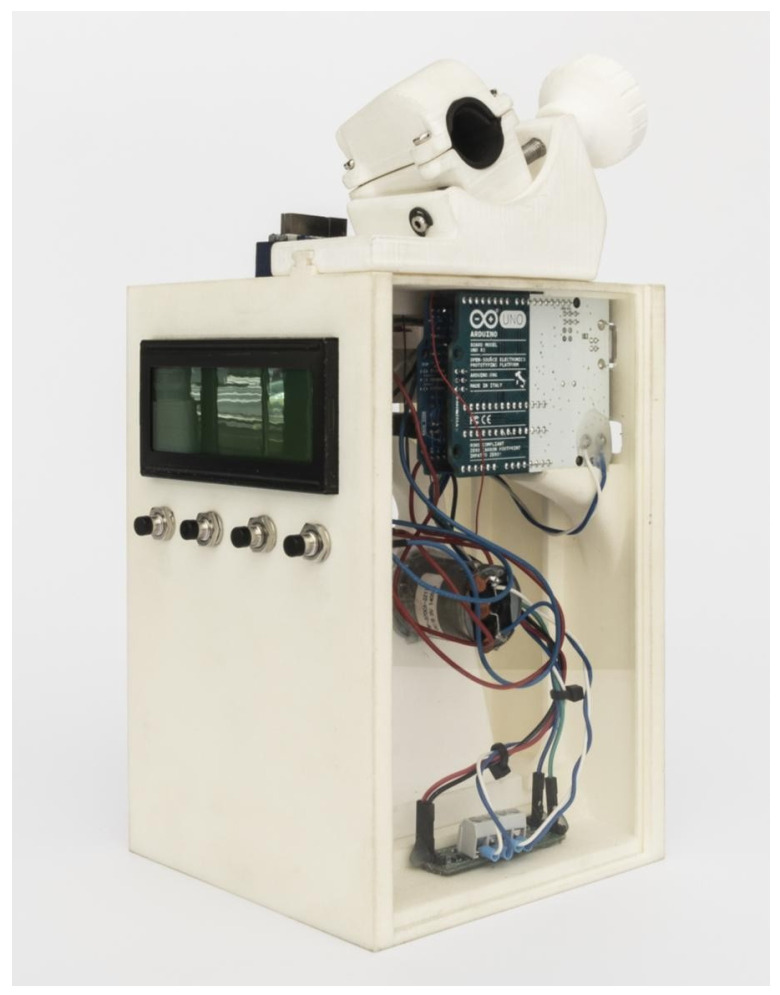
Design of prototype based on 2D/3D CAE (computer-aided engineering) (Midas FX, Brunleys, Milton Keynes, UK) software and parts of the hardware of the cyclic fatigue test device.

Cyclic fatigue experiments were carried out using a custom-made device (utility model patent number ES1219520) that informs us about the behavior of the files during use. The “Filebreaker” device guarantees the correct operation and reproducible characteristics. It was designed using 2D/3D computer-aided (CAD/CAE) software (Midas FX+^®^, Brunleys, Milton Keynes, UK) and then built using 3D printing (ProJet^®^ 6000. 3D Systems©, Rock Hill, SC, USA) (Figure 1).

**Figure 2:**
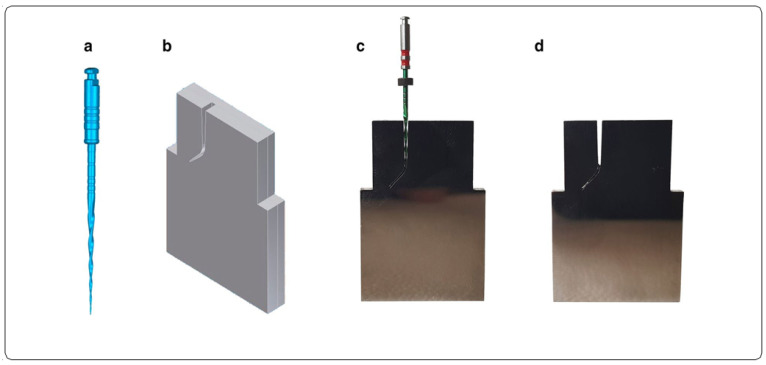
Design of artificial root canal. (**a**) Stereolithography (STL) file of the endodontic rotary file; (**b**) STL file of the artificial root canal; (**c**) endodontic rotary file in intimate contact with the artificial root canal and (**d**) artificial root canal manufactured by electrical discharge machining (EDM).

To design the artificial root canal, the reciprocating files were subjected to a Skyscan1176 microcomputerized tomography scan (Bruker-MicroCT, Kontich, Belgium) (Figure 2a), and thus, a stereolithographic (STL) file was obtained with which to design the canal (Figure 2b). It was designed with a curvature of 60° according to the Schneider measurement technique and a radius of curvature of 3 mm using 2D/3D CAD/CAE software (Midas FX+^®^, Brunleys). All of this allowed for intimate contact between the walls of the artificial root canal and the files (Figure 2b). The artificial root canal was fabricated by molybdenum wire EDM (Cocchiola SA, Buenos Aires, Argentina).

Reciprocating files were randomly assigned to groups A and B and used until failure occurred. These files were used with a 6:1 reduction handpiece (EndoPilot^®^ endodontic handpiece) and an EndoPilot^®^ motor (Schlumbohm, Brokstedt, Germany). We cannot choose the torque and revolutions per minute (rpm) because the handpiece management software adapts the movement of the files according to their resistance and tension in the artificial root canal. This software constantly analyzes the resistance suffered by the files inside the canal. Reciprocating files randomly assigned to group C were used by a 6:1 reduction handpiece, a torque-controlled motor (EndoPilot^®^ endodontic handpiece) and an EndoPilot^®^ motor (Schlumbohm, Brokstedt, Germany).

For a precise and standardized fit of the endodontic handpiece, a holder was made by 3D scanning the handpiece (Geomagic Capture Wrap, 3D Systems©, Rock Hill, SC, USA).

Reciprocating rotary files were used in the cyclic fatigue device at a rate of 60 pecking movements/min according to a previous study [22]. Special high-flow synthetic oil (Singer All-Purpose Oil; Singer Corp., Barcelona, Spain) was used to reduce friction between the walls of the artificial canal and the files.

Fracture of the files was detected with a light-dependent resistance (LDR) sensor (Ref.: C000025, Arduino LLC^®^, Ivrea, Italy), which was located at the apex of the canal. This sensor quantifies the amount of light entering through the apical end of the simulated canal, which is emitted by a high-brightness white light-emitting diode (LED) (20,000 mcd) (Ref.: 12.675/5/b/c/20k, Batuled, Coslada, Spain). The data from the LDR sensor (Ref.: C000025, Arduino LLC^®^) were conditioned by a processor (Arduino UNO Rev.3, Arduino LLC^®^, Ivrea, Italy) to detect values from 0 (endodontic rotary instrument inside the artificial root canal device) to 1024 (endodontic rotary instrument outside the artificial root canal).

Once the file fails, the LDR sensor detects it, and the software stores the time that it took to fail and the test parameters. Sensor values were displayed in real time on a liquid crystal display (LCD) that was housed in the prototype frame. All files were used until fracture occurred, and the time to failure in seconds, the number of cycles of in-and-out motions and the tip length of the fractured files were measured and recorded. The experiment was carried out at a room temperature of 23.9 °C.

### 2.3. Statistical Tests

Statistical analysis was performed with the software: SAS v9.4, SAS Institute Inc., Cary, NC, USA. Statistical decisions were made using a value of 0.05 as the level of significance. The Kolmogorov–Smirnov test was carried out to examine whether the sample followed a normal distribution. The results obtained were analyzed with the ANOVA method, and a general linear model (GLM) was fitted to evaluate the interaction between the file type and movement. In two-to-two comparisons, the *p*-values were adjusted using the Tukey method to correct type I error. A Weibull model was adjusted for time as a function of the type of file and movement.

## 3. Results

**Figure 3:**
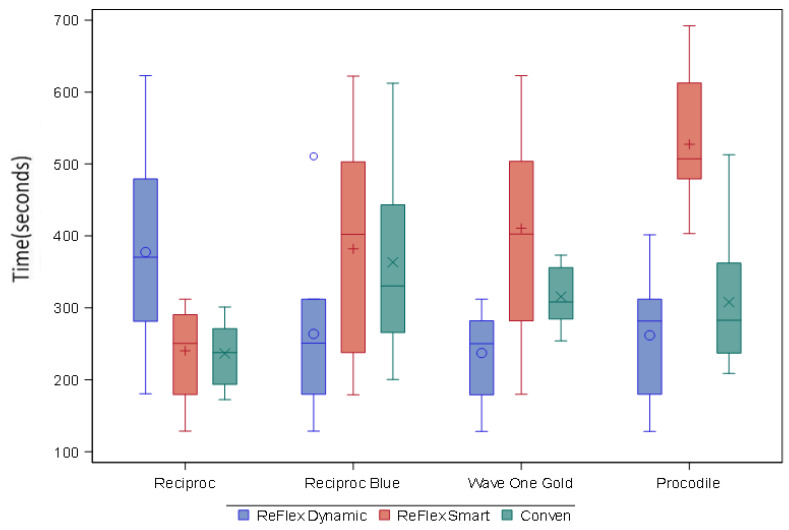
Graph of time of fracture of different system files and movements.

**Figure 4:**
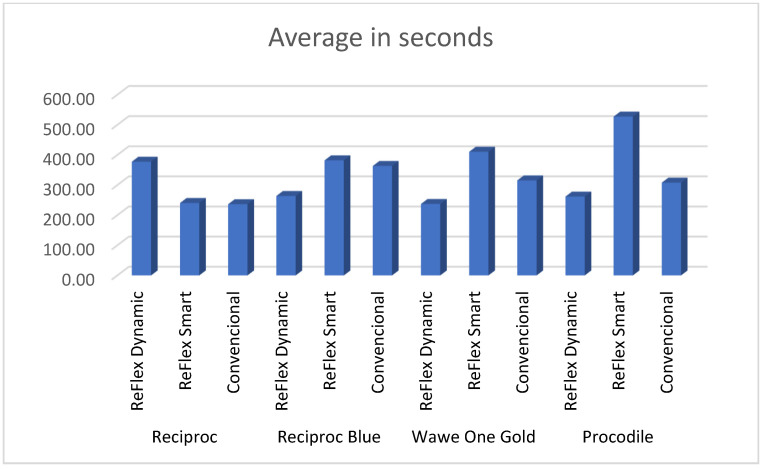
Average in seconds of different systems and movements.

The results of the mean differences are presented in Figure 3 and Figure 4.

In the statistical analysis, significant differences were observed between types of files, between movements, and between the interactions between the type of file and movement. The results of this interaction are presented in Table 1 and Table 2. Reciproc shows statistically significant differences between Reflex Dynamic and Reflex Smart and between Reflex Dynamic and conventional. Reciproc Blue shows statistically significant differences between ReFlex Smart and ReFlex Dynamic. Wave One Gold shows statistically significant differences between ReFlex Smart and Reflex Dynamic, and Procodile shows statistically significant differences between ReFlex Smart and ReFlex Dynamic and ReFlex Smart and conventional.

In the detailed comparison between the different types of files with the same movement, statistically significant differences were observed between types of files with the ‘ReFlex Dynamic’ and ‘ReFlex Smart’ movements (Table 3). Table 4 shows two-to-two comparisons. Regarding the comparison between files with the same movement, in ReFlex Dynamic, there were only statistically significant differences between Reciproc and Wave One Gold, with the longest time to failure observed for Reciproc, followed by Procodile, Reciproc Blue and Wave One Gold.

With the ReFlex Smart movement, there were statistically significant differences between Reciproc Blue and Reciproc, Wave One Gold and Reciproc, Procodile and Reciproc, and Reciproc Blue and Wave One Gold, and the longest time to failure was observed for Procodile, followed by Wave One Gold, Reciproc Blue and Reciproc.

In the conventional reciprocating movement, there were statistically significant differences between Reciproc Blue and Reciproc, and the longest time to failure was obtained with Reciproc Blue, followed by Wave One Gold, Procodile and Reciproc.

In the two-to-two comparison between all files and all movements, there were statistically significant differences (*p* < 0.005) between Procodile ReFlex Smart with all Reciproc movements and that with conventional movements, between Reciproc Blue with ReFlex Dynamic and that with conventional movements, and between Wave One Gold with ReFlex Dynamic and that with the conventional and Dynamic movements from Procodile. There was also a statistically significant difference between Wave One Gold ReFlex Smart and conventional Reciproc.

**Figure 5:**
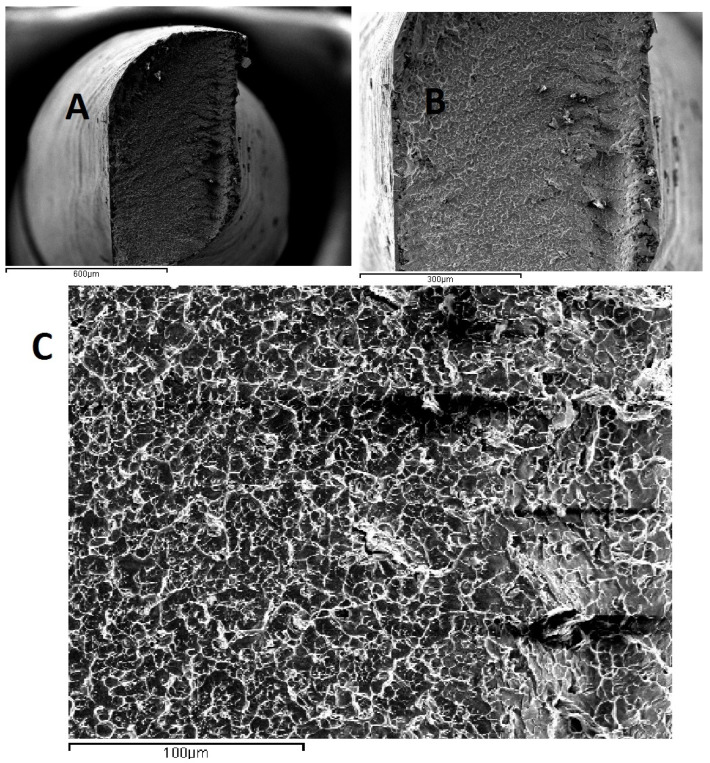
SEM photographs of the fracture site and type of fracture in Reciproc file. Crack at 45° and ductile fracture ((**A**): 100×; (**B**): 200×: (**C**): 500×).

In Figure 5, SEM photographs of the fracture site and the type of fracture are reported for the Reciproc^®^ file. The beginning of the crack at 45° and a ductile fracture can be seen.

**Figure 6:**
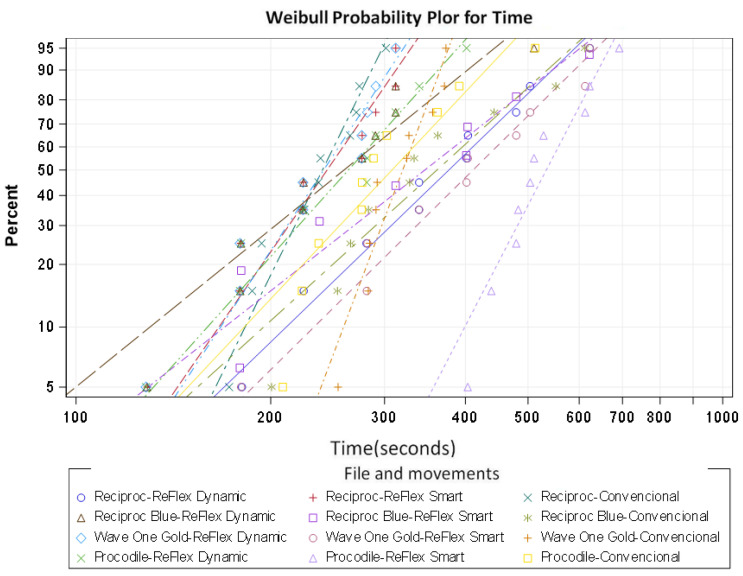
Weibull probability plot of the time to failure.

In the Weibull model, we observed that the file that behaved best with the associated movement was Procodile with Reflex Smart movement (Figure 6).

## 4. Discussion

NiTi rotary systems have changed the concept of endodontics, but currently, one of the biggest problems is the fracture of rotary instrumentation systems, since these fractured instruments can cause adverse effects on the tooth by preventing the effective disinfection of the root canal. Some of the causes that can affect the fracture of the instruments range from the number of uses, the skill of the operator, the technique and sequence of use, the anatomy of the root canal that we have to instrument, the stress to which we subject the instruments, etc. To mitigate this problem, systems have been improving, and their characteristics have been evolving, such as the design, the heat treatment to which they are subjected, the cross-section and, in recent times, also the movement.

As we can see in the results, the ReFlex Dynamic and ReFlex Smart movements increase the cyclic fatigue strength of reciprocating rotary systems compared to their conventional reciprocating movements.

It has been shown in the literature that most file fractures are caused by torsion but also by bending or cyclic fatigue. Sattapan et al. conducted a study in which they verified the causes of instrument fractures after use [23]. One of the most important factors is the curvature of the canal, since the instruments are subjected to many forces, and it is known that the cyclic fatigue strength increases when the radius and curvature of the canal decrease, that is, when the curve is smaller. In addition, the diameter of the file also influences the cyclic fatigue strength [24]. Pruett et al. concluded that the curve radius variable had an influence and that the steeper the curve, the more that the stress increased and the cyclic fatigue decreased; they also proposed the radius of curvature as an independent variable in studies to thus standardize studies a little more [25]. Topçuoğlu et al. carried out a study with artificial conduits with curvature angles of 45° and 60° to evaluate cyclic fatigue strength, and in the study, they concluded that with a curvature angle of 45°, there were no statistically significant differences, but with a curvature angle of 60°, there were [26]. This led us to use a 60° bend angle in our study. It is difficult to compare the cyclic fatigue strength between different rotary instrument systems because there are many variables that can affect the resistance of these system, such as the alloy, the iconicity or thickness and the cross-section, so it is necessary to try to standardize studies of cyclic fatigue. Cyclic fatigue can be analyzed in two different ways. One is a static method, where the file is constantly rotating inside the artificial root canal, and the handpiece does not make any movement. It can also be evaluated using a dynamic model, where the file constantly rotates while moving axially back and forth; in this way, the clinical conditions of the use of files where the file is not statically stopped are also better reproduced [27]. The FileBreaker prototype used in this study allowed us to make a dynamic model by enabling the standardization of the movement and axial speed of the handpiece. Speed, torque and the number of cycles to fracture could not be controlled in the study due to the EndoPilot^®^ handpiece software adapting to the tension and stress placed on the file.

Regarding the alloy, rotary systems have several crystalline structures: those of conventional NiTi alloy (Procodile^®^) and NiTi M-Wire have an austenitic crystalline structure; those of NiTi CM-Wire alloy have a martensitic crystalline structure (Wave One Gold^®^); and finally, NiTi systems have austenitic and martensitic crystal structures. Reciproc^®^ has a mixed austenite plus R-phase [28]. Systems with a higher martensitic phase improve some properties, such as shape memory and fracture resistance [29]. Sanchez et al. compared the cyclic fatigue strength between files with the same characteristics but with different heat treatments (ProTaper Universal F2^®^, ProTaper Gold F2^®^, ProTaper Next X2^®^ and ProFile Vortex Blue^®^) in an artificial root canal with an apical diameter of 250 µm, an angle of curvature of 60° and a radius of curvature of 5 mm, and the results indicated that the files with heat treatment were more resistant to cyclic fatigue, and when comparing ProTaper Universal with ProTaper Gold, which are identical files, but changing the heat treatment, the rest of the factors that can affect the result were eliminated since the variable of the alloy was isolated. This study leads us to confirm that the heat treatment increases the cyclic fatigue strength [30].

Almeida et al. compared the cyclic fatigue strength between Reciproc and Reciproc Blue, also isolating the heat treatment variable, as they were files with the same section and taper. The study showed that Reciproc Blue was more resistant to cyclic fatigue than Reciproc, and this was attributed to the heat treatment [31]. Scott et al. evaluated the cyclic fatigue strength of EdgeFile X1^®^, Wave One Primary^®^ and Wave One Gold Primary^®^ reciprocating systems to determine the influence of the alloy and heat treatment and showed that systems with heat-treated alloys such as Gold had higher strength to cyclic fatigue than the traditional ones with M-Wire [32].

In our study based only on movement, with a conventional reciprocating movement, Reciproc Blue (363.47 s) was the most resistant file to cyclic fatigue; it had a statistically significant difference (*p* < 0.005) from Reciproc (236.69 s), which led us to confirm that the heat treatment does directly affect cyclic fatigue. In addition, these two files have the same section and taper characteristics; after Reciproc Blue, the most resistant were Wave One Gold (315.74 s) and Procodile (308.07 s), but these have lower tapers, and Wave One Gold also has a different design, which may affect these results.

In conclusion, looking only at the alloy and smart movement results, the ReFlex Dynamic Reciproc movement (377.58 s) was superior to the rest, although there were no statistically significant differences, except between Reciproc and Wave One Gold (237.37 s). With the ReFlex Smart Procodile movement (527.43 s), it was much higher than the rest, with statistically significant differences from the rest of the files. With the ReFlex Smart Reciproc Blue movement (382.20 s), the results also showed *p* < 0.005 compared to Reciproc (240.52 s). We could not compare the alloys directly, as there were more factors that could influence the results, and we were not able to isolate only the alloy factor.

Al-Obaida et al. compared five heat-treated reciprocating systems in single- and double-bend conduits and reported that, regardless of the alloy, the italic S cross-section was the most resistant to cyclic fatigue [33], which is the section possessed by Reciproc, Reciproc Blue and Procodile. Kenskin et al. carried out a study in which they tested Reciproc Blue R25, Wave One Primary and Reciproc R25 and showed that Reciproc Blue had a significantly higher cyclic fatigue strength than the rest, and Wave One Gold obtained higher values than Reciproc for cyclic fatigue strength [34]. This is consistent with the results of our study, in which Reciproc Blue was more resistant to cyclic fatigue with conventional motion, and Wave One Gold was superior to Reciproc.

Arias et al. evaluated the influence of the cross-section by comparing Reciproc and Wave One with M-Wire alloy and showed that Reciproc had a higher cyclic fatigue strength due to its section design [35]. This is probably due to the contact of the cut surface with the root canal and the ability to remove debris.

Grande et al. analyzed the design of the file in terms of the cyclic fatigue strength of two conventional NiTi rotary systems (Mtwo^®^ and ProTaper^®^) and reported that Mtwo obtained better results than ProTaper, concluding that at the point of maximum tension, the volume of the metal could affect cyclic fatigue strength and that the larger the volume of the metal, the lower its cyclic fatigue strength. Therefore, the present study concludes that the italic S cross-section has a lower metallic mass, which influences the cyclic fatigue strength, increasing it [36]. This italic S section is present in the Reciproc, Reciproc Blue and Procodile systems and can be a differentiating factor from Wave One Gold.

DiNardo et al. conducted a study comparing reciprocating movements between ReZiflow^®^ and Wave One Gold^®^. Both files were used with the Wave One Gold movement indicated by the manufacturer, and the results showed that Reziflow was more resistant to cyclic fatigue than Wave One Gold. When using the same movement, the difference in cyclic fatigue strength could be due to the design of the cross-section or the alloy, but obtaining the best results for Reziflow, composed of conventional NiTi, indicates that the design of the cross-section has more weight than alloying or heat treatment [37]. With this study, it can be affirmed, based only on the cross-section of the instruments, that the italic S sections of Reciproc, Reciproc Blue and Procodile have greater cyclic fatigue strength than the offset parallelogram section that Wave One Gold has with any of the movements, both conventional and ReFlex Dynamic and Smart, because they are able to affect the metallic mass of the cross-section since the italic S section has less mass. Although Wave One Gold has statistically higher values than Reciproc with the ReFlex Smart movement, this greater cyclic fatigue strength cannot be said to be only due to the section, since values in which Wave One Gold is superior, such as heat treatment, have an influence, in addition to the smaller taper of the files.

There have been numerous studies about cyclic fatigue with different devices in dynamic and static tests [34,38,39]. Dynamic tests better represent, or at least are closer to, the real working conditions of the clinician [40]. Moreover, in this type of study, the time until the instrument fractures is greater when compared to that obtained in static studies, in which the stress is concentrated in a constant area [41]. The FileBreaker device was selected for this study because it represents a dynamic and reproducible model with the same conditions.

Temperature is a very important factor when it comes to NiTi variations in martensitic or austenitic phases. The experiment was carried out at room temperature, which is a limitation since the body temperature is 37 °C, and there are phase variations depending on the temperature, but it was not possible to standardize the sample if we did not keep the external temperature stable.

There are no studies in the literature on smart motions with which to compare the results of this study. However, based on the results, we can say that, in terms of the comparison between movements, all files obtained better results with smart motions than with conventional ones. ReFlex Smart was the movement with the longest time to failure in Reciproc Blue, Wave One Gold and Procodile, probably due to the double reciprocating movement that it performs, moving twice to the left when the handpiece software detects forces or tension somewhere in the instrument, which increases cyclic fatigue strength. Procodile (527.25 s) was the file that obtained the highest cyclic fatigue strength with this movement, followed by Wave One Gold, and it is possible to infer that the heat treatment has less influence on the cyclic fatigue strength than the cross-section and the metallic mass, since Procodile (without heat treatment, with the italic S section and the taper core variable) achieved better statistically significant results than the rest of the files.

In the two-to-two comparison between all files and all movements, we can also see the greater cyclic fatigue strength of the Procodile ReFlex Smart file compared to the rest of the files and movements, which indicates that the movement factor can be another variable to take into account to increase the cyclic fatigue strength of our rotary instrumentation systems.

A limitation of the present study was not including other evaluations since only resistance to cyclic fatigue was assessed, so it is recommended to evaluate this in future studies, especially clinical studies that can evaluate the results of root canal treatment while taking into account the system instrumentation, in addition to assessing the torsional fracture of the new movements.

## 5. Conclusions

Within the limitations of this study, it is concluded that the use of smart motion increased the resistance of the file system. Reciproc obtained greater cyclic fatigue strength with the ReFlex Dynamic movement than with ReFlex Smart and conventional motion. Reciproc Blue, Wave One Gold and Procodile obtained higher cyclic fatigue strength with the ReFlex Smart movement than with the rest. The ReFlex Smart movement increased the cyclic fatigue strength of reciprocating rotary systems and the file, and the most resistant movement to cyclic fatigue was Procodile ReFlex Smart. Nevertheless, further research is needed to determine the influence of these novel reciprocating movements on the cyclic fatigue resistance of heat-treated manufactured NiTi reciprocating systems.

## Figures and Tables

**Table 1:** Comparison between interactions between type of file and movement (* = *p* value < 0.05).

File	*F* Value	*p*-Value	
Reciproc	6.15	0.0029	*
Reciproc Blue	3.86	0.0240	*
Wave One Gold	7.19	0.0012	*
Procodile	19.22	<0.001	*

**Table 2:** Two-to-two comparison between interactions between type of file and movement (* = *p* value < 0.05).

File	Movement	Movement	*p*-Value	
Reciproc	ReFlex Dynamic	ReFlex Smart	0.0094	*
	ReFlex Dynamic	Conventional	0.0074	*
	ReFlex Smart	Conventional	0.9961	
Reciproc Blue	ReFlex Dynamic	ReFlex Smart	0.0296	*
	ReFlex Dynamic	Conventional	0.0801	
	ReFlex Smart	Conventional	0.9118	
Wave One Gold	ReFlex Dynamic	ReFlex Smart	0.0007	*
	ReFlex Dynamic	Conventional	0.2051	
	ReFlex Smart	Conventional	0.1000	
Procodile	ReFlex Dynamic	ReFlex Smart	<0.001	*
	ReFlex Dynamic	Conventional	0.5734	
	ReFlex Smart	Conventional	<0.001	*

**Table 3:** Comparison between different types of files with the same movement (* = *p* value < 0.05).

Movement	*F* Value	*p*-Value	
ReFlex Dynamic	3.76	0.0129	*
ReFlex Smart	13.28	<0.001	*
Conventional	2.61	0.0550	

**Table 4:** Two-to-two comparison between different types of files with the same movement (* = *p* value < 0.05).

Movement	Type of File	Type of File	*p*-Value	
ReFlex Dynamic	Reciproc	Reciproc Blue	0.0682	
	Reciproc	Wave One Gold	0.0144	*
	Reciproc	Procodile	0.0614	
	Reciproc Blue	Wave One Gold	0.9377	
	Reciproc Blue	Procodile	1.0000	
	Wave One Gold	Procodile	0.9497	
ReFlex Smart	Reciproc	Reciproc Blue	0.0131	*
	Reciproc	Wave One Gold	0.0018	*
	Reciproc	Procodile	<0.001	*
	Reciproc Blue	Wave One Gold	0.9249	
	Reciproc Blue	Procodile	0.0104	*
	Wave One Gold	Procodile	0.0577	
Conventional	Reciproc	Reciproc Blue	0.0329	*
	Reciproc	Wave One Gold	0.3145	
	Reciproc	Procodile	0.4057	
	Reciproc Blue	Wave One Gold	0.7245	
	Reciproc Blue	Procodile	0.6215	
	Wave One Gold	Procodile	0.9983	

## Data Availability

The datasets used and/or analyzed during the current study are available from the corresponding author on reasonable request.
